# Development of an Efficient Analytical Method for the Extraction and Analysis of Biocide Contents from the Textile Test Specimens on LC-DAD

**DOI:** 10.1155/2020/3047961

**Published:** 2020-03-30

**Authors:** Muhammad Jahangir, Uzman Khan

**Affiliations:** Department of Chemistry, Government College University, Lahore, Pakistan

## Abstract

Biocides are frequently used in the manufacturing of textiles that are in direct contact with human skin. Recently regulated biocides do not have validated methods for testing; so, their presence cannot be estimated in the consumer products. Hence a rapid method was developed for the separation and quantitative analysis of biocide contents (2-methyl-4-isothaizolin (MIT), 5-chloro-2-methyl-4-isothaizolin-3-one (CIT), 2-octo-4-isothaizolin-3-one (OIT), and 5-chloro-2-(2,4-dichlorophenxy) phenol (triclosan)) from the textile test specimens. Test specimens were extracted with methanolic sonication and purified by centrifugation and filtration. Biocide contents were separated at C18 column with 0.4% acetic acid: methanol (1 : 1 v/v) under isocratic mode and detected at 280 nm wavelength. Pretreatment factors such as extraction solvent, extraction method, dilution ratio, and extraction time were optimized initially and plotted calibration curve showed regression (*r*^2^ ≥ 0.9995) in the range of 1.0–5.0 mg L^−1^. Recoveries were between 95% and 108% with the relative standard deviation ≤ 4%. Limits of detection (LODs) were between 0.06 mg L^−1^ and 0.12 mg L^−1^ and limits of quantification (LOQs) were between 0.21 mg L^−1^ and 0.38 mg L^−1^. From the results, conclusion was made that the method can achieve the purpose of quantitative detection and the analysis of real test specimens verified the reliability of this method.

## 1. Introduction

Biocides are active substances that reduce harmful effect, intended to destroy, prevent the action of, or otherwise exert a controlling effect on any harmful biological organism by chemical or biological mode of action instead of just physical or mechanical action. Biocidal Products Directive 98/8/EC was in force from May 13, 2000, until August 31, 2013. After that, it was reviewed and replaced with the biocidal product regulation (BPR EU 528/2012) [1, 2]. Biocides are used in a variety of consumer products like rubber, polymerized material, paper, textile, and leather finishes [3]. For much demanding functionality of the products like wrinkle resistance, water repelling, fade resistance, and resistance to microbial invasion, there has been upsurge interest in apparel technology all over the world. Since the garments are in direct contact with human body, so the development of antimicrobial textile finish is highly indispensable and relevant [4–9]. Several challenges have been created for apparel researchers due to increasing global demand in textile as cotton fabrics provide ideal environment for microbial growth. There is an increasing demand on global scale for textile fabrics with antimicrobial finishes. Several antimicrobial agents as quaternary ammonium compounds, triclosan, and recently nanosilver biocide contents are available for textile finishing [8, 9]. They are synthetic in nature which creates environmental problems [10]. Biocides are widely used in everyday life as active ingredients in a variety of textile, pharmaceutical, and personal care products; due to this reason they have received increasing attention as emerging contaminants [11, 12]. Contamination of the receiving environment is due to the extensive use and high emission of these biocides [13, 14]. Biocides also have been reported in various environmental media especially in wastewater treatment plants (WWTPs). In 19 Australian WWTPs at average concentrations of 142 ng L^−1^ for effluents and 5.58 mg/kg for biosolids, triclosan has been reported. In the literature, a variety of extraction and instrumental analysis methods for the biocide contents are present. But current literature study shows that systematic screening of various classes of biocides in textile matrices has not been performed yet. From the available literature, it is revealed that only few studies have been explored on screening methods of banned antimicrobial agents on textile materials [15–19]. There is strong need for consolidated data and progressive research for screening and testing methods on antimicrobial finished product of textile. In the present work, we discuss the determination of biocide contents in different models of the finished consumer goods related to textile for women, men, and children commercialized in Pakistan or produced for export. The developed method was applied to the analysis of these target biocide contents in the textile test specimens.

## 2. Experimental

### 2.1. Chemicals and Solvents

All the organic solvents and analytical grade solid chemicals were purchased from local suppliers. MIT, CIT, OIT, and triclosan were from Chem Service; methanol HPLC grade and glacial acetic acid analytical grade were from Lab-Line supplier in Lahore city.

### 2.2. Preparation of Stock and Working Standard Solutions

For the preparation of 1000 mg L^−1^ MIT, CIT, OIT, and triclosan stock standard solutions, 10.00 mg of each of MIT, CIT, OIT and triclosan standards was weighed, respectively, and made up to mark in separate 10 mL volumetric flasks with methanol. Working standard solutions of 100 mg L^−1^ were prepared by pipetting 500 *μ*L of 1000 mg L^−1^ MIT, CIT, OIT, and triclosan stock solutions in separate 5 mL flasks and diluting each volumetric flask up to mark with methanol.

Note that CIT/MIT stock solution is relatively more stable. So, for preparation of stock solutions, CIT/MIT stock solution was prepared collectively.

### 2.3. Preparation of Calibration Standard Solutions

Five-point calibration standard solutions of 1.0, 2.0, 3.0, 4.0, and 5.0 mg L^−1^ of CIT, MIT, OIT, and triclosan each were prepared separately from 100 mg L^−1^ working standard solutions.

### 2.4. Test Specimen's Collection

Commercial textile products were collected from textile process industries and retail stores in Pakistan from April to June 2018.

### 2.5. Instrumentation

LC chromatographic system (Shimadzu) LC -20AT liquid chromatograph equipped with a SPD-M20A UV DAD detector, auto sampler SIL-20AHT, column oven CTO-20A, degasser unit DGU-20A5R, and quaternary pump LC 20AT (serial no. L20114811607) was used for quantitative analysis. Lab Solutions software was used (Shimadzu Corporation, version 5.71).

## 3. Instrument Conditions for MIT, CIT, OIT, and Triclosan

The chromatographic column Hypersil C18 (250 mm × 4.6 mm × 5 *μ*m) was used. 0.4% acetic acid and methanol (50 : 50/v: v) were used as mobile phases at 280 nm wavelength. 30°C was the column oven temperature and 30 *μ*L was the injection volume at 1.0 mL min^−1^ flow rate in isocratic mode. Total run time at LC instrument was 12.0 min.

### 3.1. Test Specimen Preparation

From the textile consumer goods, the test specimen was taken randomly from the different parts of the test specimen. If the textile test specimen was single colored and homogeneous, the test specimen was cut into pieces of approximately 5 mm × 5 mm and mixed. If the test specimen was multicolored or with pattern, the test specimen was collected according to proportion of color, cut into pieces of approximately 5 mm × 5 mm, and mixed well.

**Figure 1:**
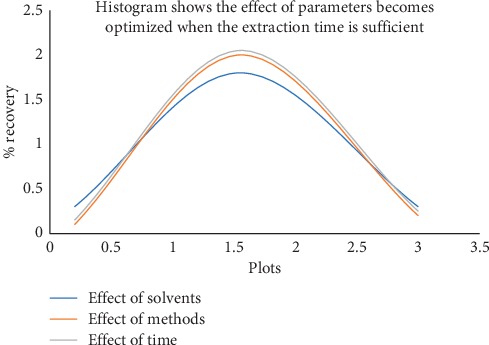
Extraction effect of different extraction solvents, methods, and time on biocide contents (mg L^−1^) in textile test specimens A, B, and C spiked with known amount of analyte 3.0 mg L^−1^.

Three test specimens, A, B, and C, were selected as blank matrix and spiked with 3.0 mg L^−1^ of each pure standard of biocides. These three test specimens were analyzed using different extraction solvents, methods, and time. Recovery was best in methanol using ultrasonic method at 30 min. Hence, methanol was chosen as extraction solvent, ultrasonic as extraction method, and 30 min as extraction time (results were summarized in Figure 1 and Tables “[Supplementary-material supplementary-material-1][Supplementary-material supplementary-material-1]” in supplementary data).

### 3.2. Test Specimen Extraction

We accurately weighed 5.00 ± 0.01 g test specimen on an analytical balance Mettler Toledo (model: ML 204/01) and transferred the test specimen into a reagent bottle; 80 mL methanol was added to the 100 mL reagent bottle and sonicated at 50°C for 30 min. Test specimens were totally extracted within 30 min sonication. After 30 min time, reaction vessel was cooled down to room temperature within 2 min. Extract was concentrated to about 2 mL at 55°C by rotary vacuum evaporator. The concentrated extract was diluted to 5 mL with methanol in a volumetric flask, filtered with 0.45 *μ*m glass wool filter in 1.5 mL GC vial.

### 3.3. Forced Degradation Study for MIT, CIT, OIT, and Triclosan

Accelerated degradation studies were performed on MIT, CIT, OIT, and triclosan. Acidic degradation study was performed by taking the 5 mL methanolic solution of biocide in 1.5 mL 0.1 N HCl at ambient temperature for 1 hour. Alkaline degradation study was performed by taking the 5 mL biocide solutions in 1.5 mL 0.1 M NaOH at ambient temperature for 1 hour. Thermal degradation was performed by exposing 5 mL biocide solutions at 80°C for three days. Oxidative degradation study was performed by taking the 5 mL biocide solutions in 30% v/v H_2_O_2_ at ambient temperature for 1 hour. Photolytic degradation study was performed by exposing the biocide solutions in UV-light at 320 nm wavelength for three days.

### 3.4. Instrumental Analysis by LC/DAD

Prior to running any batch of test specimens on instrument, the following parameters were performed.

LC/DAD instrument conditions were set as (A) instrumental conditions for MIT, CIT, OIT, and triclosan. Calibration curve was established for each analyte using peak area vs. concentration. The coefficient of linear regression (*r*) was ≥0.995. Calibration standard check solution (CC) 3.0 mg L^−1^ was injected to the instrument; recovery of calibration check was within range of 80%–120%. Prior to running any batch of test specimens, method blank and specimen blank were injected to the instrument to check any contamination. Sensitivity check 1.0 mg L^−1^ was analyzed for instrument response examination. Laboratory quality control 3.0 mg L^−1^ and specimen spike 3.0 mg L^−1^ were injected to the instrument to check for experimental recovery. After all these interim checks test specimen extract was injected, the presence of target analyte was identified based on retention time and comparison of the UV spectrum, and background correction was made, with characteristic wavelength in a reference UV spectrum.

The relative retention time of the test specimen component was within the ±0.01 retention time units of the relative retention time of the standard components and the peak maxima/minima of the test specimen component were within ±1 nm of that in the reference spectrum. Specimen spike was injected per batch of test specimen to check for experiment recovery. If the response ratio (RR) for any quantitation wavelength exceeds the initial calibration range of the LC/DAD instrument, the test specimen extract was diluted for required range and was reanalyzed.

### 3.5. Result Calculation

The biocide contents of the test specimens were calculated according to the following equation and rounded off to two decimal places.

Total concentration of analyte in test specimen (mg kg^−1^) = concentration of analyte in solution (mg L^−1^) × dilution factor (mL)/test specimen weight (g).

## 4. Results and Discussion

Analytical method was validated prior to the introduction into routine analyses.

### 4.1. Specificity

Matrix blank, reagent blank, and pure standards were analyzed to observe the effect of possible interference of any matrix or reagent on analytes and chromatographic technique (results were summarized in [Supplementary-material supplementary-material-1] of supplementary data).

### 4.2. Accuracy

A blank and a test specimen was spiked with pure standard of concentrations 1.0 mg L^−1^, 3.0 mg L^−1^, and 5.0 mg L^−1^ and these individually prepared replicates were analyzed at each concentration level. Recovery of these replicates was within the range of 100 ± 10% (results were summarized in Figure 1 and Table “[Supplementary-material supplementary-material-1]” in supplementary data).

### 4.3. Precision

A test specimen solution was prepared, containing the target level of analyte. 10 replicates were made from this test specimen solution according to the final method procedure and analysis was performed in the subsequent six days. The relative standard deviation was within the range of 1.8%–2.8% for same day and 2.4%–3.9% for 6 days (results were summarized in Table 1).

### 4.4. LOD and LOQ

LOD and LOQ of the proposed method was calculated by preparing a blank solution and spiked solutions with progressively decreasing known concentrations of each analyte. The developed method was used to analyze these solutions. By evaluating the minimum concentrations for each analyte that can be detected and quantified with accuracy (for the LOD signal-to-noise ratio of 3 : 1 and for the LOQ 10 : 1), the LOD and LOQ were determined for the proposed method. The limits of detections (LODs) were between 0.06 mg L^−1^ and 0.12 mg L^−1^ and limits of quantification (LOQs) were between 0.21 mg L^−1^ and 0.38 mg L^−1^ for target analytes (results were summarized in Table 1).

### 4.5. Linearity and Working Range

A five-point calibration curve was drawn (points were 1.0, 2.0, 3.0, 4.0, and 5.0 mg L^−1^). Curve was linear with regression in the range of 0.9995 to 0.9999. 1.0 mg L^−1^–5.0 mg L^−1^ was the working range of the curve. Each concentration level was prepared and analyzed three times (results were summarized in Figures m and n in supplementary data).

### 4.6. Selectivity

The selectivity of the proposed LC-DAD method was observed by preparing mixture of analytes with commonly occurring interferences found in textile test specimens and the percent recovery of each analyte in the presence of interferences was calculated (results were summarized in Figure 1 and Table “[Supplementary-material supplementary-material-1]” in supplementary data).

### 4.7. Robustness

The robustness of the proposed method was evaluated by intentionally changing the chromatographic parameters. Mobile phases were changed from 0.4% citric acid: methanol (50 : 50) to 45 : 55. Flow rate was changed from 1 mL/min to 0.9 mL and 1.1 mL/min. Column oven temperature was varied as ±3°C. Deviation in results was ±1% only. Hence, it was concluded that varying the conditions had no appreciable effect on analytes. The results of the robustness study were summarized in Tables 2 and 3 (results were given for MIT and CIT only).

### 4.8. Stability

In the presence of the other analyte in the solution, the stability of each analyte was determined by calculating the percent deviation of the results obtained after three days' period and it was compared with the data at the start time. The deviation of each analyte was observed which was less than 2% in the three days' period.

### 4.9. Degradation Study of Analytes

Accelerated degradation of the analytes in the mixture was performed to evaluate the specificity of the proposed method. The analytes were subjected to forced degradation as acidic, basic, thermal, and oxidative conditions. The test specimens treated with HCl showed considerable degradation for the analytes. The biocide contents were found to be degraded up to 4–10% in acidic condition, whereas in the case of alkaline degradation, it was observed that around 3–12% of the biocide contents were degraded and 6–14% biocide contents were degraded under thermal degradation condition. In oxidative degradation, it was found that around 7–13% of the biocide contents were degraded. Major degradation was observed in photolytic condition which was 12–34%. The chromatographic peaks of the degradation products were in good condition and were well separated from the analyte peaks under all the stress conditions and this separation showed the specificity of the method in the presence of the degradation products. Under the same conditions, a mixture of possible interfering substances (placebo) was also analyzed to evaluate their interfering effect. The absence of chromatographic peaks showed the specificity of the method.

**Figure 2:**
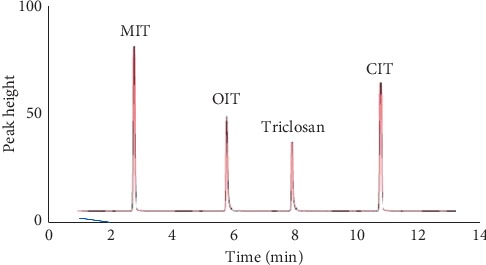
LC peaks of biocides at 3 mg L^−1^ concentration in methanol.

Peak resolution was good for all the analytes in detected test specimens and all the analytes elute before 12 min time. MIT elutes at 2.75 min, OIT at 5.85 min, triclosan at 7.85 min, and CIT at 10.65 min (results were summarized in Figure 2). Quantification of test specimens was performed by external standard method. Biocide contents leaching out from test specimens were in the range of 2.86 mg L^−1^ to 75.56 mg L^−1^, which were summarized in Table 4 (only positively tested test specimens were summarized). A total of 135 test specimens were analyzed for biocide contents in the three months' period.

Test specimens A, B, and C were used as blank matrix for analyzing the parameters as extraction solvent, extraction method, and extraction time for biocide contents from textile test specimens. Test specimens A, B, and C were spiked with 3.0 mg L^−1^ biocide contents (CIT, OIT, MIT, and triclosan). Different solvents as methanol, acetonitrile, water, water/methanol 1 : 1, and acetonitrile/water 1 : 1 were used to extract these biocide contents. Recovery was better in methanol as compared to other solvents. Ultrasonic bath and centrifuge and water both with shaker were used as extraction methods. Ultrasonic method was better than centrifuge and water bath method. 10, 20, 30, 40, 50, and 60 min time were used to extract biocide contents. 30 min time was adequate to extract total amount of biocides form textile test specimen (results were summarized in Figure 1 and Tables “[Supplementary-material supplementary-material-1][Supplementary-material supplementary-material-1]” in supplementary data).

### 4.10. Test Specimens Screened for MIT, CIT, OIT, and Triclosan

The lowest value for these biocides was 2.86 mg kg^−1^ and the highest value was 75.56 mg kg^−1^. Test specimen D contained 4.38 mg kg^−1^ MIT, 8.95 mg kg^−1^ CIT, 10.99 mg kg^−1^ OIT, and 12.44 mg kg^−1^ triclosan; test specimen E contained 10.83 mg kg^−1^ CIT and 3.92 mg kg^−1^ OIT; test specimen F contained 6.53 mg kg^−1^ OIT and 2.93 mg kg^−1^ triclosan; test specimen G contained 3.83 mg kg^−1^ MIT and 2.86 mg kg^−1^ CIT; test specimen H contained 10.45 mg kg^−1^ MIT; test specimen I contained 25.35 mg kg^−1^ CIT and 2.99 mg kg^−1^ triclosan; and test specimen J contained 75.56 mg kg^−1^ MIT and 17.43 mg kg^−1^ triclosan (results were summarized in Table 4).

## 5. Conclusions

The suitability of solvent extraction for the determination of four biocide contents (MIT, CIT, OIT, and triclosan) from the textile test specimens was determined. The validated method was effectively used to analyze the textile test specimens, maintaining high sensitivity and selectivity for target analytes. Test specimens were extracted with methanolic sonication, separated on C18 column, and detected with a diode array detector (DAD). Under the optimized conditions, good linearity (*r*^2^ ≥ 0.9995) and recovery (95%–108%) were observed for target analytes. From the results, conclusion was made that the method can achieve the purpose of quantitative detection. So, this method can be used for the quantitative detection of biocide contents from the commercial textile test specimens. The proposed method could be a useful tool to control the safety of the textile test specimens. This method will be helpful for biomonitoring and tracking of these chemicals associated with human exposure through direct contact and use.

## Figures and Tables

**Table 1:** Regression equation, the limit of detection (LOD), the limit of quantification (LOQ), and relative standard deviation (RSD) for MIT, CIT, OIT, and triclosan.

Target	Regression equation	Correlation coefficient	LOD (mg L^−1^)	LOQ (mg L^−1^)	RSD in the same day^a^ %	RSD in 6 days^a^ %
MIT	*Y* = 0.8360X − 0.0598	0.9999	0.11	0.36	1.8	2.4
CIT	*Y* = 0.6460X − 0.0308	0.9998	0.08	0.28	2.5	3.8
OIT	*Y* = 0.4180X − 0.0309	0.9997	0.12	0.38	2.8	3.9
Triclosan	*Y* = 0.3601X − 0.0245	0.9995	0.06	0.21	1.9	2.9

*Y* = peak area, *X* = mean concentration (mg L^−1^), linear range = 1.0–5.0 mg L^−1^, and ^a^*n* = 10.

**Table 2:** Results of robustness study on MIT.

Condition	Assay, %	Retention time, minutes	Number of theoretical plates	Tailing	Resolution
0.4% citric acid: methanol (50 : 50)	100.18	2.506	18340	0.96	10.36
0.4% citric acid: methanol (55 : 45)	99.42	2.485	18076	1.18	6.05
0.4% citric acid: methanol (45 : 55)	100.60	3.365	18897	1.09	12.28
Flow rate, 1.1 mL min^−1^	99.78	2.455	18225	1.06	9.85
Flow rate, 0.9 mL min^−1^	100.33	2.535	17432	1.20	10.70
Column oven temperature 33	100.40	2.475	16234	1.34	9.65
Column oven temperature 27	100.38	2.511	18954	1.08	10.26

**Table 3:** Results of robustness study on CIT.

Condition	Assay, %	Retention time, min	Number of theoretical plates	Tailing	Resolution
0.4% citric acid: methanol (50 : 50)	100.12	9.815	17834	0.94	10.12
0.4% citric acid: methanol (55 : 45)	99.63	9.735	18123	1.13	5.90
0.4% citric acid: methanol (45 : 55)	100.74	11.285	18435	1.06	12.22
Flow rate, 1.1 mL min^−1^	99.85	9.785	18034	1.04	9.63
Flow rate, 0.9 mL min^−1^	100.54	9.945	17380	1.15	10.56
Column oven temperature 33	100.30	9.810	16554	1.24	9.56
Column oven temperature 27	100.25	9.925	18765	1.02	10.06

**Table 4:** Results of detectable test specimens analyzed on LC/DAD mg kg^−1^.

Test specimens	Test specimen description	MIT mg kg^−1^	CIT mg kg^−1^	OIT mg kg^−1^	Triclosan mg kg^−1^
A	Black denim fabric	BDL	BDL	BDL	BDL
B	Blue denim fabric	BDL	BDL	BDL	BDL
C	Black fabric + foam	BDL	BDL	BDL	BDL
D	Black foam + fabric	4.38	8.95	10.99	12.44
E	Black denim fabric	BDL	10.83	3.92	BDL
F	Black denim pant	BDL	BDL	6.53	2.93
G	Black denim pant	3.83	2.86	BDL	BDL
H	Brown fabric	10.45	BDL	BDL	BDL
I	Yellow fabric	BDL	25.35	BDL	2.99
J	Black denim pant	75.56	BDL	BDL	17.43

## Data Availability

The supplementary data is attached herewith.

## Supplementary Materials

Supplementary Materials Table a: extraction effect of different extraction solvents on biocide contents (mg L^−1^) in textile test specimens spiked with known amount of analyte 3.0 mg L^−1^. Figures a–c: extraction effect of different extraction solvents on biocide contents (mg L^−1^) in textile test specimens A, B, and C spiked with known amount of analyte 3.0 mg L^−1^. Table b: extraction effect of different extraction methods on biocide contents in textile test specimens spiked with known amount of analyte 3.0 mg L^−1^. Figures d–f: extraction effect of different extraction methods on biocide contents (mg L^−1^) in textile test specimens A, B, and C spiked with known amount of analyte 3.0 mg L^−1^. Table c: effect of extraction time on extraction efficiencies of biocide contents in textile test specimens spiked with known amount of analyte 3.0 mg L^−1^. Figures i–k: effect of extraction time on extraction efficiencies of biocide contents (mg L^−1^) in textile test specimens A, B, and C spiked with known amount of analyte 3.0 mg L^−1^. Table d: recoveries of biocide contents from textile test specimens. Figure l: recoveries of biocide contents from textile test specimens A, B, and C in mg L^−1^. Figure m: five-point calibration curve (data from instrument). Figure n: five-point calibration curve (data from instrument).
