# Supplementary material for: Development of an Efficient Analytical Method for the Extraction and Analysis of Biocide Contents from the Textile Test Specimens on LC-DAD
Source: J Anal Methods Chem. 2020 Mar 30;2020:3047961. doi: 10.1155/2020/3047961 (PMC7149327; doi:10.1155/2020/3047961)
Supplement: Supplementary Materials — Table a: extraction effect of different extraction solvents on biocide contents (mg L−1) in textile test specimens spiked with known amount of analyte 3.0 mg L−1. Figures a–c: extraction effect of different extraction solvents on biocide contents (mg L−1) in textile test specimens A, B, and C spiked with known amount of analyte 3.0 mg L−1. Table b: extraction effect of different extraction methods on biocide contents in textile test specimens spiked with known amount of analyte 3.0 mg L−1. Figures d–f: extraction effect of different extraction methods on biocide contents (mg L−1) in textile test specimens A, B, and C spiked with known amount of analyte 3.0 mg L−1. Table c: effect of extraction time on extraction efficiencies of biocide contents in textile test specimens spiked with known amount of analyte 3.0 mg L−1. Figures i–k: effect of extraction time on extraction efficiencies of biocide contents (mg L−1) in textile test specimens A, B, and C spiked with known amount of analyte 3.0 mg L−1. Table d: recoveries of biocide contents from textile test specimens. Figure l: recoveries of biocide contents from textile test specimens A, B, and C in mg L−1. Figure m: five-point calibration curve (data from instrument). Figure n: five-point calibration curve (data from instrument). [file 3047961.f1.docx]

Supplementary data

Table a. Extraction effect of different extraction solvents on biocide contents (mg L^-1^) in textile test specimens spiked with known amount of analyte 3.0 mg L^-1^

| Test specimen | Target | Spiked amount 3.0 mg L^-1^ | | | | |
| --- | --- | --- | --- | --- | --- | --- |
|  |  | Recovery (mg L^-1^) in Methanol | Recovery  (mg L^-1^) in  Acetonitrile | Recovery  (mg L^-1^) in  Water | Recovery  (mg L^-1^) in  Water/Methanol  (1:1) | Recovery  (mg L^-1^) in  Acetonitrile/Water  (1:1) |
| A | MIT | 2.89 | 2.25 | 2.45 | 2.82 | 2.84 |
|  | CIT | 3.16 | 2.18 | 2.67 | 3.32 | 3.35 |
|  | OIT | 3.23 | 2.67 | 2.26 | 2.68 | 2.64 |
|  | Triclosan | 3.34 | 2.34 | 2.38 | 2.85 | 2.72 |
| B | MIT | 2.97 | 2.62 | 2.67 | 2.99 | 3.19 |
|  | CIT | 3.02 | 3.34 | 3.18 | 3.13 | 3.26 |
|  | OIT | 2.96 | 2.77 | 2.78 | 3.12 | 2.63 |
|  | Triclosan | 2.96 | 2.78 | 2.88 | 2.85 | 2.91 |
| C | MIT | 3.05 | 2.21 | 8.87 | 2.77 | 3.03 |
|  | CIT | 2.86 | 2.65 | 2.66 | 2.76 | 2.73 |
|  | OIT | 3.15 | 2.83 | 2.76 | 2.78 | 3.17 |
|  | Triclosan | 3.06 | 3.35 | 3.45 | 2.85 | 3.24 |

Figure a.

Figure b.

Figure c.

Figure a, b and c. Extraction effect of different extraction solvents on biocide contents (mg L^-1^) in textile test specimens A, B and C spiked with known amount of analyte 3.0 mg L^-1^

Table b. Extraction effect of different extraction methods on biocide contents in textile test specimens spiked with known amount of analyte 3.0 mg L^-1^

| Test specimen | Target | Spiked amount 3.0 mg L^-1^ | | |
| --- | --- | --- | --- | --- |
|  |  | Recovery  (mg L^-1^) using  Ultrasonic | Recovery  (mg L^-1^) using  Centrifuge | Recovery  (mg L^-1^) using  Water both with shaker |
| A | MIT | 3.15 | 3.63 | 2.85 |
|  | CIT | 3.25 | 2.51 | 2.67 |
|  | OIT | 3.12 | 2.78 | 2.95 |
|  | Triclosan | 3.17 | 2.88 | 3.12 |
| B | MIT | 3.14 | 3.11 | 2.83 |
|  | CIT | 2.97 | 2.87 | 2.64 |
|  | OIT | 3.01 | 2.89 | 2.71 |
|  | Triclosan | 2.89 | 2.87 | 2.81 |
| C | MIT | 2.99 | 2.99 | 2.92 |
|  | CIT | 2.88 | 2.87 | 2.73 |
|  | OIT | 2.98 | 3.14 | 3.17 |
|  | Triclosan | 2.77 | 2.56 | 2.15 |

Figure d.

Figure e.

Figure f.

Figure d, e and f. Extraction effect of different extraction methods on biocide contents (mg L^-1^) in textile test specimens A, B and C spiked with known amount of analyte 3.0 mg L^-1^

Table c. Effect of extraction time on extraction efficiencies of biocide contents in textile test specimens spiked with known amount of analyte 3.0 mg L^-1^

| Test specimen | Target | Spiked amount 3.0 mg L^-1^ | | | | | |
| --- | --- | --- | --- | --- | --- | --- | --- |
|  |  | Recovery  mg L^-1^  in 10 min | Recovery  mg L^-1^  in 20 min | Recovery  mg L^-1^  in 30 min | Recovery  mg L^-1^  in 40 min | Recovery  mg L^-1^  in 50 min | Recovery  mg L^-1^  in 60 min |
| A | MIT | 3.23 | 3.05 | 3.06 | 3.06 | 3.14 | 3.08 |
|  | CIT | 2.61 | 2.88 | 2.97 | 2.96 | 3.05 | 3.06 |
|  | OIT | 3.15 | 3.23 | 3.16 | 3.16 | 3.14 | 3.16 |
|  | Triclosan | 3.22 | 3.16 | 3.15 | 3.25 | 3.22 | 3.17 |
| B | MIT | 2.74 | 2.87 | 2.98 | 2.97 | 2.99 | 2.93 |
|  | CIT | 3.13 | 3.19 | 3.05 | 3.05 | 3.05 | 3.15 |
|  | OIT | 2.75 | 2.83 | 2.99 | 2.95 | 2.97 | 2.98 |
|  | Triclosan | 2.72 | 2.76 | 2.97 | 2.95 | 2.96 | 2.97 |
| C | MIT | 2.64 | 2.78 | 2.99 | 2.89 | 2.78 | 2.88 |
|  | CIT | 3.21 | 3.35 | 3.14 | 3.16 | 3.28 | 3.22 |
|  | OIT | 3.16 | 3.11 | 2.98 | 2.97 | 3.10 | 3.18 |
|  | Triclosan | 3.14 | 3.16 | 3.06 | 3.11 | 3.13 | 3.10 |

Figure i.

Figure j.

Figure k.

Figure i, j and k Effect of extraction time on extraction efficiencies of biocide contents (mg L^-1^) in textile test specimens A, B and C spiked with knoawn amount of analyte 3.0 mg L^-1^

Table d. Recoveries of biocide contents from textile test specimens

| Test specimen | Spike level | Target | Quantity detected mg L^-1^ | Quantity Spiked mg L^-1^ | Quantity Found mg L^-1^ | Recovery % |
| --- | --- | --- | --- | --- | --- | --- |
| Blank | Low | MIT | BDL | 1 | 0.97 | 97.00 |
|  |  | CIT | BDL | 1 | 0.96 | 96.00 |
|  |  | OIT | BDL | 1 | 0.95 | 95.00 |
|  |  | Triclosan | BDL | 1 | 1.08 | 108.00 |
|  | Medium | MIT | BDL | 3 | 3.13 | 104.33 |
|  |  | CIT | BDL | 3 | 3.06 | 102.00 |
|  |  | OIT | BDL | 3 | 3.01 | 100.33 |
|  |  | Triclosan | BDL | 3 | 2.98 | 99.33 |
|  | High | MIT | BDL | 5 | 4.94 | 98.80 |
|  |  | CIT | BDL | 5 | 4.97 | 99.40 |
|  |  | OIT | BDL | 5 | 5.05 | 101.00 |
|  |  | Triclosan | BDL | 5 | 5.13 | 102.60 |
| A | Low | MIT | BDL | 1 | 1.06 | 106.00 |
|  |  | CIT | BDL | 1 | 1.05 | 105.00 |
|  |  | OIT | BDL | 1 | 1.07 | 107.00 |
|  |  | Triclosan | BDL | 1 | 0.98 | 98.00 |
|  | Medium | MIT | BDL | 3 | 3.03 | 101.00 |
|  |  | CIT | BDL | 3 | 3.06 | 102.00 |
|  |  | OIT | BDL | 3 | 2.95 | 98.33 |
|  |  | Triclosan | BDL | 3 | 2.96 | 98.67 |
|  | High | MIT | BDL | 5 | 4.94 | 98.80 |
|  |  | CIT | BDL | 5 | 4.98 | 99.60 |
|  |  | OIT | BDL | 5 | 5.12 | 102.40 |
|  |  | Triclosan | BDL | 5 | 5.06 | 101.20 |
| B | Low | MIT | BDL | 1 | 0.98 | 98.00 |
|  |  | CIT | BDL | 1 | 0.97 | 97.00 |
|  |  | OIT | BDL | 1 | 0.96 | 96.00 |
|  |  | Triclosan | BDL | 1 | 1.09 | 109.00 |
|  | Medium | MIT | BDL | 3 | 3.12 | 104.00 |
|  |  | CIT | BDL | 3 | 3.08 | 102.66 |
|  |  | OIT | BDL | 3 | 3.01 | 100.33 |
|  |  | Triclosan | BDL | 3 | 2.97 | 99.00 |
|  | High | MIT | BDL | 5 | 4.92 | 98.40 |
|  |  | CIT | BDL | 5 | 4.97 | 99.40 |
|  |  | OIT | BDL | 5 | 5.09 | 101.80 |
|  |  | Triclosan | BDL | 5 | 5.13 | 102.60 |
| C | Low | MIT | BDL | 1 | 1.10 | 110.00 |
|  |  | CIT | BDL | 1 | 1.07 | 107.00 |
|  |  | OIT | BDL | 1 | 1.06 | 106.00 |
|  |  | Triclosan | BDL | 1 | 0.95 | 95.00 |
|  | Medium | MIT | BDL | 3 | 3.03 | 101.00 |
|  |  | CIT | BDL | 3 | 3.08 | 102.66 |
|  |  | OIT | BDL | 3 | 2.90 | 98.33 |
|  |  | Triclosan | BDL | 3 | 2.96 | 96.66 |
|  | High | MIT | BDL | 5 | 4.84 | 96.80 |
|  |  | CIT | BDL | 5 | 4.93 | 98.60 |
|  |  | OIT | BDL | 5 | 5.12 | 102.40 |
|  |  | Triclosan | BDL | 5 | 5.21 | 102.20 |

BDL = Below Detection Limit

Figure l. Recoveries of biocide contents from textile test specimen’s A, B and C in mg L^-1^

Table f. Interim checks

| Perameter | Abrivation | Quantity spiked (mg L^-1^) | Quantity detected  (mg L^-1^)^e^ |
| --- | --- | --- | --- |
| Method blank | MB | BDL | BDL |
| Specimen blank | SB | BDL | BDL |
| Sensitivity check | SC | 1.0 | 1.03 |
| Lab quality check | LQC | 3.0 | 2.95 |
| Specimen spike | SS | 3.0 | 2.98 |
| Calibration standard check | CSC | 3.0 | 3.01 |

BDL: Below detection limit, ^e^: three replicate measurement


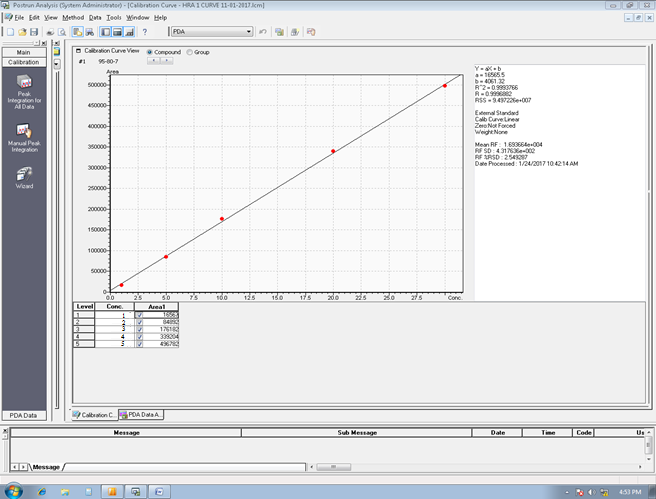


Figure m. Five-point calibration curve (data from instrument)

Figure n. Five-point calibration curve (data from instrument)
